# External misattribution of internal thoughts and proneness to auditory hallucinations: the effect of emotional valence in the Deese–Roediger–McDermott paradigm

**DOI:** 10.3389/fnhum.2013.00351

**Published:** 2013-07-09

**Authors:** Mari Kanemoto, Tomohisa Asai, Eriko Sugimori, Yoshihiko Tanno

**Affiliations:** ^1^Department of Cognitive and Behavioral Science, Graduate School of Arts and Science, The University of TokyoTokyo, Japan; ^2^Department of Psychology, Chiba UniversityChiba, Japan; ^3^Department of Psychology, Yale UniversityNew Haven, CT, USA

**Keywords:** auditory hallucination-like experience, DRM paradigm, emotional valence, source monitoring, schizophrenia, thought insertion

## Abstract

Previous studies have suggested that a tendency to externalize internal thought is related to auditory hallucinations or even proneness to auditory hallucinations (AHp) in the general population. However, although auditory hallucinations are related to emotional phenomena, few studies have investigated the effect of emotional valence on the aforementioned relationship. In addition, we do not know what component of psychotic phenomena relate to externalizing bias. The current study replicated our previous research, which suggested that individual differences in auditory hallucination-like experiences are strongly correlated with the external misattribution of internal thoughts, conceptualized in terms of false memory, using the Deese–Roediger–McDermott (DRM) paradigm. We found a significant relationship between experimental performance and total scores on the Launay–Slade Hallucination Scale (LSHS). Among the LSHS factors, only vivid mental image, which is said to be a predictor of auditory hallucinations, was significantly related to experimental performance. We then investigated the potential effect of emotional valence using the DRM paradigm. The results indicate that participants with low scores on the LSHS (the low-AHp group in the current study) showed an increased discriminability index (*d*′) for positive words and a decreased *d*′ for negative words. However, no effects of emotional valence were found for participants with high LSHS scores (high-AHp group). This study indicated that external misattribution of internal thoughts predicts AHp, and that the high-AHp group showed a smaller emotional valence effect in the DRM paradigm compared with the low-AHp group. We discuss this outcome from the perspective of the dual-process activation-monitoring framework in the DRM paradigm in regard to emotion-driven automatic thought in false memory.

## Introduction

Schizophrenia is a severe psychiatric disorder characterized by positive (e.g., auditory hallucinations and delusional beliefs) and negative symptoms (e.g., anhedonia). Auditory hallucinations are one of the most common symptoms of schizophrenia, occurring in approximately 60–80% of affected individuals (Wing et al., 1974; Slade and Bentall, 1988; Ditman and Kuperberg, 2005), and is used as a diagnostic criterion for the illness (APA, 1994) (Waters et al., 2012). Beavan and Read (2010) observed that studies employing strict definitions of hearing a voice that has no corresponding external stimulus while in a conscious, wakeful state reported that 2–4% of the general population has experienced auditory hallucinations (Read et al., 2006; Beavan and Read, 2010). However, if the definition of voices is broadened to include those experienced while in an altered state of consciousness, such as hypnogogic and hypnopompic hallucinations or drug-induced states (or banal misinterpretations of ambiguous noises, such as hearing one's name called in a public place), then up to 84% of the general population have experienced hearing voices (Millham and Easton, 1998). Auditory hallucinations within the general population have been found to be associated with the same risk factors that predict psychotic disorders (Van Os et al., 2009), which is consistent with the hypothesis of a symptom continuum from nonclinical through clinical populations (Van Os et al., 2000). It might then be expected that auditory hallucinations within the general population are driven by cognitive mechanisms that are similar to those underlying auditory hallucinations in psychotic patients. Therefore, studies investigating the cognitive mechanisms underpinning the proneness to auditory hallucinations (AHp) in nonclinical samples are likely to be relevant to the early detection of psychosis (Johns and Van Os, 2001).

### Self-monitoring approach to auditory hallucinations

Among the positive symptoms of schizophrenia, auditory hallucinations, thought insertion, and delusions of control are called passive phenomena because the patient perceives his/her actions and thoughts as originating from external stimuli (Asai et al., 2008). Frith (1987) first suggested that passive phenomena may be attributable to a self-monitoring disorder (self-monitoring theory). Self-monitoring theory predicts that, by nature, normal people may be able to monitor their own actions or thoughts, whereas those who suffer from passive phenomena may lack this ability and thereby misattribute their own actions or thoughts to external stimuli. Many experimental methods have been proposed to investigate self-monitoring theory. For example, experiments using the “on-line self-monitoring task” revealed that people experiencing hallucinations were more likely than normal healthy people to regard their own voices as belonging to someone else when feedback of their own voices was altered on-line (e.g., Johns et al., 2006). “Source-monitoring tasks” revealed that people experiencing hallucinations were more likely than normal healthy people to misattribute the source of words uttered by themselves to the experimenter (e.g., Brébion et al., 2000). Similar results have been found in non-clinical populations using the on-line self-monitoring task (Asai and Tanno, 2012) and the source-monitoring task (Sugimori et al., 2011a).

### The DRM paradigm and misattributions of internal thoughts

Auditory hallucinations may occur without any actual audible output (McGuire et al., 1996a,b; Jones and Fernyhough, 2007), suggesting that auditory hallucinations could be one form of thought insertion (Morrison et al., 1995). Sommer et al. (2010) proposed a theoretical framework in which verbal auditory hallucinations have two essential components: audibility, including verbal imagery or thoughts, and alienation, and that the latter component might lead to the symptom of thought insertion. Moreover, neuroimaging studies have already suggested that patients with schizophrenia demonstrate not only impaired verbal self-monitoring, but also failure to activate cortical areas underlying normal monitoring of inner speech and verbal imagery (McGuire et al., 1996a,b).

Although it is important to investigate the external misattribution of internal thoughts to understand the mechanisms underpinning auditory hallucinations, the factor of “thought” was overlooked in previous behavioral studies of speech because it is difficult to directly investigate it via an on-line self-monitoring task. Memory tasks should be useful for investigating the external misattribution over thought. That is, the memory “I was thinking about that at that time” can serve as a potential indicator of on-line self-monitoring (e.g., the sense of agency) of thought (“I am thinking about that now”) because contemporaneous judgments about the origins of thoughts are difficult to measure. Daprati et al. (2003) proposed a link between memory and the sense of agency on the basis of experiments investigating the effect of agency on both explicit and implicit memory traces (Franck et al., 2000; Daprati et al., 2005). Furthermore, in order to directly demonstrate the relationship between sense of agency (on-line-sense of enactment) and recalled judgment about enactment (the judgment of “I did it”), we previously focused on speech (Sugimori et al., 2011a). Through these experiments, the probability has been suggested that memory judgment of enactment might be based on the on-line sense of enactment.

The Deese–Roediger–McDermott, or DRM, paradigm (Roediger and McDermott, 1995) has also been used to investigate the external misattribution of “internal thoughts” or “inner speech” (Sugimori et al., 2011b).

The DRM paradigm was originally proposed to measure false memories (Roediger and McDermott, 1995). In this paradigm, participants are presented with a series of words (e.g., *door, glass, pane, shade, ledge, sill, house, open, curtain, frame, view, breeze, sash, screen*, and *shutter*) that are strongly associated with an unidentified target item, referred to as the critical lure (in this case, *window*; Howe et al., 2010). Roediger and McDermott (1995) and others (e.g., Deese, 1959) have found that normal healthy controls often falsely recall or recognize the critical lure in subsequent testing, and that such false memories are often associated with high levels of confidence.

The dual-process activation-monitoring framework was developed to explain how semantic related false memories occur in the DRM paradigm (McDermott and Watson, 2001). According to this framework, when we hear list items during encoding or retrieval, we think about the critical non-presented associate (critical lure) and made it more easily accessible through spreading activation in the semantic network. In this sense, the DRM paradigm, in which the critical lures falsely recalled by subjects are their externalized inner associations, is also the measure of self-monitoring error (e.g., externalizing) of inner thought or inner speech.

When considering the dual-process nature of the DRM paradigm, however, we speculate that false memories of critical lure were caused by a self-monitoring error only when the semantic network activation is similar for each participant. Indeed, as patients with schizophrenia generally experience the particular symptom of a thought disorder known as loose associations, some studies have suggested that they may not be able to activate the semantic network for the critical lure, resulting in findings of no difference in the false-alarm rates of patients and controls (Elvevag et al., 2004; Moritz et al., 2004; Lee et al., 2007). On the other hand, in a sample of recruited non-clinical individuals, Sugimori et al. (2011b) did not find the semantic activation deficit in participants who were highly prone to auditory hallucinations (high-AHp) and could extract only the effect of external misattribution. Sugimori et al. (2011b) therefore concluded that the higher false-alarm rates found for the high-AHp group represent a tendency towards the external misattribution of one's own automatic internal thoughts.

### Emotional valence in auditory hallucinations

A classical perspective in the assessment of schizophrenia emphasizes derogatory content in auditory hallucinations (Fish, 1967). Others have maintained that auditory hallucinations incorporate affirmative and/or benevolent content (Miller et al., 1993; Chadwick et al., 2000). Previous studies have investigated external misattribution bias in schizophrenic and AHp subjects as modulated by the affective components of hallucination-related content (Johns et al., 2001; Larøi et al., 2004; Costafreda et al., 2008). These studies suggested that, compared with controls, schizophrenia and AHp groups were significantly more likely to produce external misattributions under the negative-word than under the neutral-word condition. Given that previous research with schizophrenic subjects viewed external attribution as a mechanism for reducing anxiety, it was not surprising that hallucination-negative content was externalized, whereas hallucination-neutral content was not (Costafreda et al., 2008). However, the effects of the emotional valence of hallucination-positive content remain unknown. Moreover, as these previous studies used either the on-line self-monitoring task or the source-monitoring task (Johns et al., 2001; Larøi et al., 2004; Costafreda et al., 2008), the negative emotional valence effect on the external misattribution of internally generated thoughts remains unclear. In this study, we used the DRM paradigm to investigate the effects of both positive and negative emotional valence on the external misattribution of internally generated thoughts.

One potential result might reflect a higher rate of false memories of the critical lure in the high-AHp group than in the low-AHp group in the negative word condition because of their increased source-monitoring errors. This result is consistent with the results of previous studies using source-monitoring and on-line self-monitoring tasks (Johns et al., 2001; Larøi et al., 2004; Costafreda et al., 2008). However, we could have expected the opposite result by using the DRM paradigm with regard to the negative emotional valence effect, because the DRM paradigm includes another complicating factor; namely, semantic network activation processing.

In healthy people, emotional valence strengthens similarity between words and increases activation of the semantic network (Kensinger, 2004). Yet it is thought that both the negative and positive symptoms of patients with schizophrenia reflect emotional deficits (Alba-Ferrara et al., 2012). For example, previous findings indicate that patients with schizophrenia are more likely to delay the processing of emotion-laden material, when compared with controls (Rockstroh et al., 2006; Seok et al., 2006). It has also been reported that schizotypal individuals were less affected by emotional priming, suggesting that this population produces fewer emotion-driven associations (Kerns, 2005). Additionally, schizophrenic patients tend to avoid negative emotional stimuli, possibly, at least in part, because this patient population presents as emotionally overwhelmed when encountering such perceived negatively associated stimuli (Aleman and Kahn, 2005; Kerns, 2005; Seok et al., 2006). Because of such emotional deficits, emotion-driven semantic activation may not occur in the high-AHp group, which can lead them not to think about the critical lure and, consequently, reduce false recollection. Therefore, we hypothesized that the false memory of critical lures, under the negative condition, occurs more frequently in the low-AHp group than in the high-AHp group. Results in support of this hypothesis suggest that the emotional DRM paradigm has utility for investigating the effect of emotional valence, not only on the external misattribution of internal thoughts, but also on the activation of the semantic network in auditory hallucination prone subjects.

### The current study

We initially hypothesized that this study would find a positive correlation between scores on auditory hallucination scales and rates of external misattribution of internal thoughts in the DRM task. Additionally, we examined potential emotional valence effects on the DRM task in terms of individual differences in AHp. We evaluated study outcomes regarding the auditory hallucination process from the perspective of self-monitoring theory, as well as from the perspective of the dual-process activation-monitoring framework for associative and/or automatic thought.

## Materials and methods

### Participants

Fifty Japanese university students were recruited. But one female was excluded due to obtaining outlying scores on the AHES-17 and the Launay–Slade Hallucination Scale (LSHS) (see next section) that would have biased the results, particularly in the correlation analyses. Thus, data from 49 participants were analyzed (mean age = 19.53; age range = 18–25 years; sex = 34 males, 15 females). Participants were tested individually and provided written informed consent before the experiments were conducted.

### Experimental task

#### Materials

We used Japanese emotional word lists that were highly likely to induce false memories of critical non-presented words within the context of the DRM paradigm (Roediger and McDermott, 1995; Takahashi, 2001; Kobayashi and Tanno, 2012). Included for use were four negative lists (e.g., *pressure, pain, suicide, war*), four positive lists (e.g., *wish, sun, nature, courtesy*), and four neutral lists (e.g., *opinion, stamp, important, clock*) taken from Kobayashi and Tanno (2012) and Takahashi (2001). Each list consisted of 15 Japanese words, whose emotional valences were investigated by Kobayashi and Tanno (2012). We added 5 unrelated neutral words to each list, and each complete list included 15 associated words and 5 non-associated words. Ten of the 15 associated words of each list were designated as to-be-learned items in the learning phase and used as targets in the test phase; the remaining 5 associated words (critical lures) and the 5 non-associated words (non-critical lures) were used as distractors in the test phase only. Free auditory-editing software was used to pre-record the 10 to-be-learned items; these items were recorded as a female voice.

We selected 5 critical lures, with their emotional valence to be consistent with that of the list, as not all 15 words had an emotional valence (Kobayashi and Tanno, 2012). Non-associated words (5 words per list, 60 words in total) were selected from the other 4 neutral lists not used in the learning phase. Their listed words were not semantically associated with the listed words used in the learning phase. As a result, 10 to-be-learned words, five critical lures, and five neutral non-critical lures were used in each trial.

#### Procedure

In the experiment, participants completed 12 trials (four positive-, four neutral-, and four negative-emotion lists), in random order, following one practice trial. Each trial included a learning phase (auditory presentation of 10 words), a filler task (simple math calculations), and a test phase (visual presentation of 20 words for old–new recognition). Participants completed the computer-based experiment (Windows XP, E-prime 2.0) in a dark, semi-soundproof booth.

In the learning phase, after participants pushed the space key, 10 words were presented in random order over headphones. The duration of each word was approximately 1200 ms, and the interval between words was 300 ms. Participants were instructed to “just carefully listen to these words.” During the filler phase, participants were instructed to complete computational math questions that were presented on computer for 1.0 min. They received feedback on the correctness of each answer and were instructed to solve as many problems as possible. During the test phase, including the old–new recognition test, 20 words were presented in random order and participants were instructed to indicate whether each word presented had been presented during the learning phase. After all questions were answered, the learning phase for the next trial was ready to start. If participants felt tired, they could rest before the learning phase of the next trial.

### Questionnaire battery

Participants were given two booklets of questionnaires and completed all questions. One booklet, administered before the experimental task, included the Beck Depression Inventory–Second Edition (BDI-II; Beck et al., 1996) to control for the influence of mood on performance of the DRM task. The remaining booklet was administered following the experimental task and included the AHES-17, the LSHS, and the Schizotypal Personality Questionnaire–Brief Form (SPQ-B; Raine and Benishay, 1995). Participants could take as much time as they needed to complete the questionnaires.

#### Auditory hallucination experience scale–brief version (AHES-17)

The AHES-17 is a 17-item self-report questionnaire scored on a five-point (1–5) Likert scale (e.g., “I heard someone's voice, but nobody was actually around”). Test–retest reliability (*r* = 0.78, *p* < 0.0001) and internal reliability (α = 0.84) were adequate, and investigation of criterion-related validity showed that the AHES-17 was strongly correlated with scales measuring the positive symptoms of schizophrenia including auditory hallucinations. Furthermore, we initially reconfirmed the reliability and factor structure of these instruments in a large sample (379 men, 234 women; mean age of 19.23, *SD* = 0.98). The AHES-17 has two factors. One is auditory hallucination-like experiences about internal speech, music, and others. The other factor is delusions or thought insertions related to auditory hallucinations (Asai et al., 2011).

#### Launay–slade hallucination scale (LSHS)

The LSHS (Bentall et al., 1989; Launay and Slade, 1981) is a 12-item self-report questionnaire scored on a five-point (1–5) Likert scale (e.g., “I often hear a voice speaking my thoughts aloud”). The LSHS measures hallucination-like experiences, including auditory hallucinations. This scale is frequently used to measure hallucinatory experiences in both clinical and nonclinical populations. According to recent research, the LSHS has two factors: one is hallucinatory experience and the other is vivid mental events (Fonseca-Pedrero et al., 2010).

#### Schizotypal personality questionnaire-brief form (SPQ-B)

The SPQ-B is a 22-item, true/false self-report questionnaire consisting of items selected from the SPQ, a 74-item self-report scale based on the diagnostic criteria for schizotypal personality disorder in the Diagnostic and Statistical Manual, Third Edition, Revised (DSM-III-R; APA, 1987). The SPQ-B has the same three-factor structure as does the SPQ and includes items such as, “Have you ever noticed a common event or object that seemed to be a special sign for you?” “Have you found that it is best not to let other people know too much about you?” and “I find it hard to communicate clearly what I want to say to people.” These test items measure cognitive, interpersonal, and disorganized factors, respectively.

#### Beck depression inventory–second edition (BDI-II)

The BDI-II, a 21-item self-report questionnaire that is one of the most widely used instruments for measuring the severity of depression, was developed based on the diagnostic criteria for depression included in the Diagnostic and Statistical Manual of Mental Disorders, Fourth Edition (DSM-IV). The fourth edition of the DSM incorporated several significant changes to the diagnostic criteria for Major Depressive Disorder. For this study, participants were asked to rate how they have been feeling for the past 2 weeks, on a four-point scale (0–3). The Japanese version of the BDI-II has been confirmed as equivalent to the original edition.

On the basis of results of our previous study (Sugimori et al., 2011b) we expected that scores on the LSHS and AHES-17 would be correlated with performance on the experimental task. We used the BDI-II to control for the influence of mood and used the SPQ-B to control for other schizophrenic-like personality traits.

### Analysis

We assessed the external misattribution of automatic and/or associative thought with the discriminability index (*d*′) based on signal detection theory (Macmillan and Creelman, 1990). This index was calculated for each participant for critical lures as follows:

$$d^{\prime }=z(\text{hits})-z(\text{falsealarms})$$

When the rate of false alarms was zero, a correction was applied such that the zero count was replaced by a count of 1/2*N*, where *N* = total hits + misses. When the rate of hits was one, a correction was applied such that the one count was replaced by a count of 1−1/2*N*, where *N* = total correct rejections + false alarms (Macmillan and Creelman, 1990). The *d*′ varies from zero, where 0 = chance performance, to 4.64, where 4.64 = perfect performance.

First, we conducted an analysis of variance (ANOVA) for each emotional condition in the experimental performance in order to investigate an emotional valence effect in the DRM paradigm regardless of individual differences. Second, to determine individual differences in the DRM paradigm, we conducted a simple correlation and multiple regression analysis between questionnaire-based traits and the *d*′-values for the critical lure. We also analyzed correlations between questionnaire-based traits and false alarms for non-critical lures to confirm that subjects had no semantic activation deficit. Semantic activation deficit was measured as a decrease in false memories of the critical lure and an increase in those of the non-critical lure as memories of items semantically associated with learned items may not be activated, whereas memories of items not semantically associated with learned items may be activated (Huron and Danion, 2002). We did not use *d*′ for non-critical lures because the false-alarm rate for non-critical lures was zero for many subjects. If external misattribution of internal thought were highly related to the factor of auditory hallucination, no other questionnaire score, such as SPQ-B or BDI-II, would predict the *d*′ of critical lures. Furthermore, we conducted a group comparison (high-AHp group vs. low-AHp group) to examine the relationship between the negative and positive emotional valence effects and auditory hallucination proneness in terms of the interaction model of the ANOVA (emotional valence effects on *d*′ × AHp groups).

## Results

### General performance in the emotional DRM paradigm

Table 1 presents the demographic data for the current and normative samples. Compared with results of our previous study (Sugimori et al., 2011b), the scores for hallucination-related traits (LSHS, AHES-17, and Cog in SPQ-B) were slightly lower in the current sample, although the distributions in each study were almost equivalent.

**Table 1:** **Demographic data of the current and normative samples**.

**Measure**	**Sample means (*SD*)**	**Norms means (*SD*)**
Launay-slade hallucination scale (LSHS)	23.7 (7.1)	26.7 (7.8)
Auditory hallucination experience scale (AHES-17)	48.5 (10.9)	51.6 (11.5)
Schizotypal personality questionnaire-Brief (SPQ-B)	8.9 (4.4)	10.2 (5.0)
Cognitive (Cog)	1.6 (2.2)	2.2 (1.9)
Interpersonal (Int)	4.3 (1.4)	4.7 (2.0)
Disorganization (Dis)	3.0 (2.0)	3.3 (3.4)
Beck depression inventory (BDI-II)	9.0 (7.1)	N.A.

Note: Norms based on the data of our previous study, Sugimori et al. (2011b) (N = 172, for each).

Regardless of emotional valence, the mean hit rate (i.e., the rate at which learned items were correctly identified as learned) was.84 (*SD* = 0.08), whereas the mean rate of false alarms (i.e., the rate at which unlearned words were incorrectly identified as learned) was 0.31 (*SD* = 0.13) in response to critical non-presented words and 0.03 (*SD* = 0.03) in response to non-critical non-presented words. Because these results meant that critical non-presented words were associated with more false alarms than were non-critical non-presented words, we confirmed that critical lures caused enough false alarms for this procedure and for the select lists used in this experiment (e.g. Dehon et al., 2010; Sugimori et al., 2011b). The calculated mean *d*′-value from these hit-and-false-alarm ratios was 1.56 (*SD* = 0.40).

**Figure 1:**
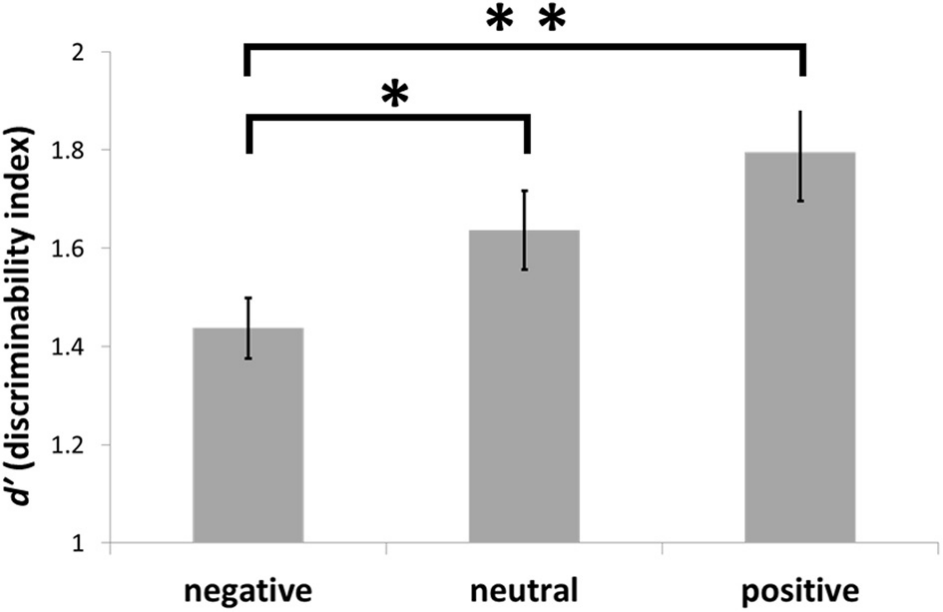
**The *d*′ under each emotional condition in the DRM paradigm.** Note: error bars mean ± 1 S.E. ^*^ < 0.05, ^**^ < 0.01.

When we examined the effect of emotional valence on *d*′, the averaged *d*′ was 1.44 (*SD* = 0.43) under the negative condition, 1.64 (*SD* = 0.56) under the neutral condition, and 1.82 (*SD* = 0.70) under the positive condition (Figure 1). A one-way ANOVA (three emotional conditions) with repeated measures was performed. The main effect of emotional conditions was significant [*F*_(2, 96)_ = 8.37, *p* < 0.001]. The *post hoc* comparison using Ryan's method revealed significant differences between negative and neutral (*p* = 0.01) and negative and positive (*p* < 0.001) conditions, indicating the presence of emotional valence effects; that is, the discriminability index, *d*′, for negative words was lower and the *d*′ for positive words was higher. In this study, the former is referred to as the “negative emotional valence” effect and the latter as the “positive emotional valence” effect. Although the emotional valence effects on false memory have not been observed in a consistent way in previous studies, the results of the current study are consistent with those of two earlier studies (see Discussion for details) with regard to the negative emotional valence effect.

### Correlation and multiple regression analysis between questionnaire scores and experimental performance

**Figure 2:**
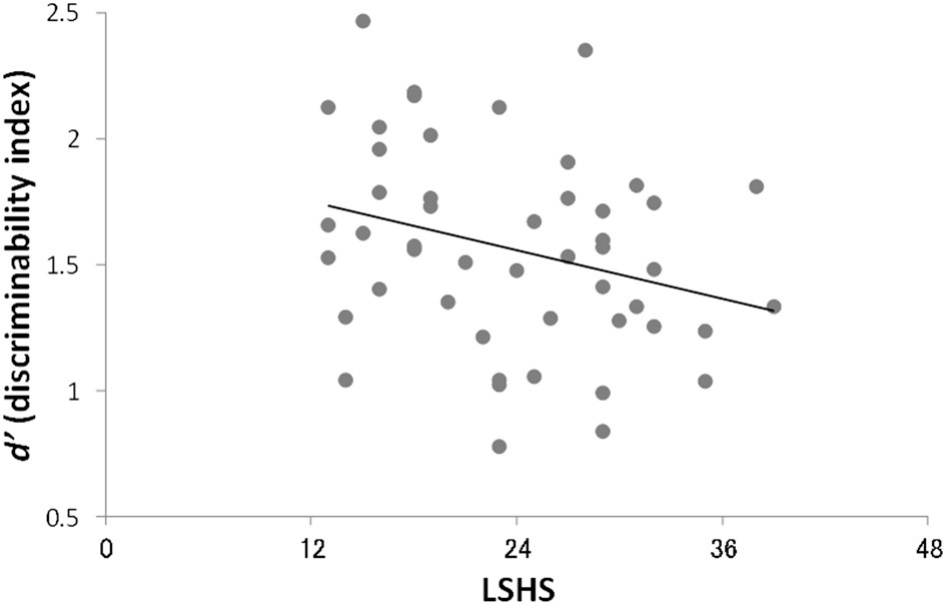
**Scatter plot between LSHS scores and total *d*′**.

**Figure 3:**
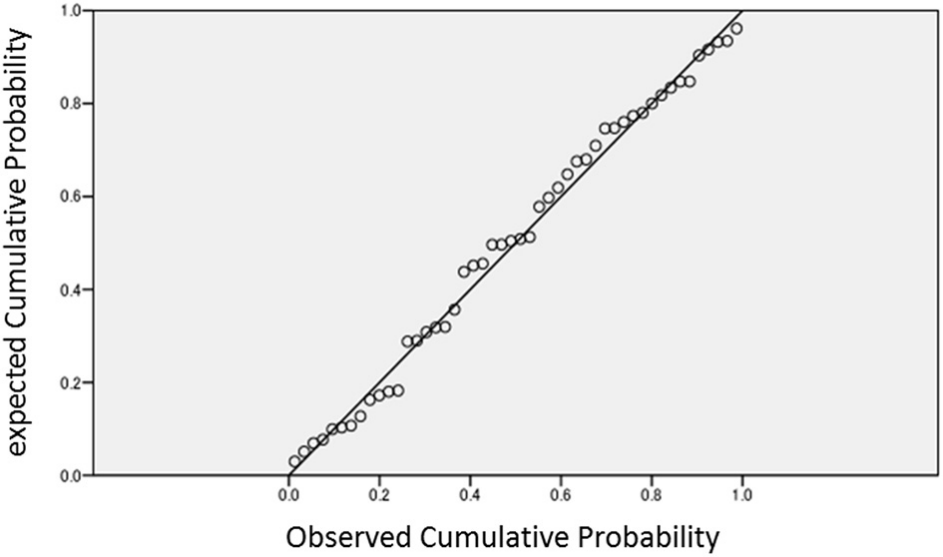
**The Normal P-P plot of Regression Standardized Residual**.

Pearson's correlation analysis revealed a significant correlation between the total *d*′ for critical non-presented words and scores on the LSHS (*r* = −0.29, *p* = 0.045) (Table 2, Figure 2). This correlation remained significant while controlling for the emotional state (i.e., scores on the BDI-II) during the experimental task (partial correlation *r* = −0.30, *p* < 0.042). Correlations between *d*′ and AHES-17 (*r* = −0.05, *p* = 0.72), and *d*′ and SPQ-B scores (*r* = −0.12, *p* = 0.43), were not significant. These results indicate that participants with higher LSHS scores showed lower discriminability for the critical lures on the old–new recognition task. When we applied multiple regression analysis to control for the possibility of making a type I error, our model showed significant amount of variance in *d*′ scores [*R*^2^ = 0.20, *F*_(6, 41)_ = 2.93, *p* = 0.02], and the LSHS, nevertheless, made a significant contribution to the explanation of the total *d*′-values (*p* = 0.003) (Table 3). We confirmed normality of residuals in order to verify whether the assumption that the residuals or error terms were normally distributed has been met (see Figure 3). The plot of residuals fits the expected pattern well enough to support a conclusion that the residuals were normally distributed.

**Table 2:** **Correlation between the experimental performances and questionnaire measures**.

	***d*′ for critical lure**				**FA for non-critical lure**			
	**Total**	**Negative**	**Neutral**	**Positive**	**Total**	**Negative**	**Neutral**	**Positive**
LSHS	−0.29^*^	−0.05	−0.29^*^	−0.29^*^	0.19	0.31^*^	0.02	0.11
AHES-17	−0.05	0.12	−0.11	−0.08	0.24	0.29^*^	0.01	0.26
SPQB	−0.12	0.12	−0.19	−0.16	0.12	0.14	0.01	0.15
Cog	0.08	0.26	−0.01	−0.05	−0.02	0.06	−0.13	0.02
Int	−0.13	0.08	−0.23	−0.13	0.11	0.10	0.12	0.02
Dis	−0.17	−0.02	0.15	−0.18	0.16	0.15	−0.02	0.29
BDI-II	0.09	0.26	−0.01	−0.01	0.20	0.18	0.25	0.03

* < 0.05.

**Table 3:** **Simultaneous regression analysis of d′ for critical lures in relation to the AHES-17, LSHS, SPQ-B, and BDI-II**.

		**Total *d*′**				
**Variable**		***B***	**S EB**	**ß**	***t***	***P***
LSHS		−0.033	0.011	−0.59	−3.12	0.00^*^
AHES-17		0.014	0.008	0.38	1.82	0.08
SPQ-B	Int	−0.021	0.027	−0.11	−0.77	0.45
	Cog	0.107	0.046	0.38	2.31	0.03^*^
	Dis	−0.101	0.037	−0.50	−2.72	0.01^*^
BDI-II		0.014	0.009	0.26	1.63	0.11

* < 0.05.

Our result was different from that of Sugimori et al. (2011b), who indicated that both the LSHS and AHES-17 were significantly correlated with performance of the DRM task and only the AHES-17 contributes significantly to the explanation of performance on the neutral DRM task. In order to further investigate which factor of the auditory hallucination-related scales could predict the performance of the DRM task, we conducted a multiple regression analysis again considering each of the 2 factors of the AHES-17 and LSHS (Fonseca-Pedrero et al., 2010; Asai et al., 2011) (Table 4). Our model showed significant amount of variance in d' scores [*R*^2^ = 0.17, *F*_(8, 39)_ = 2.23, *p* = 0.046], and only one factor of the LSHS, Vivid Mental Events, made a significant contribution to the explanation of the total *d*′-values (*p* = 0.01). Vivid Mental Events is a factor composed of six questions (e.g., “Sometimes my thoughts seem as real as actual events in my life”) and measures individual differences in participants' vividness of mental image. Although Sugimori et al. (2011b) did not discuss the factors of the AHES-17 or the LSHS, the current finding suggests that the present emotional DRM paradigm might be related to vividness of mental image rather than being directly related to auditory hallucinations itself in the non-clinical sample.

**Table 4:** **Simultaneous regression analysis of *d*′ for critical lures in relation to the each factor of AHES-17 and LSHS, and SPQ-B, BDI-II**.

		**Total d′**				
**Variable**		***B***	**S EB**	**ß**	***t***	***P***
LSHS-I (vivid mental events)		−0.043	0.016	−0.532	−2.61	0.01^*^
LSHS-II (hallucinatory experience)		−0.021	0.024	−0.162	−0.91	0.37
AHES-17-l (auditory hallucinations)		0.016	0.009	0.379	1.85	0.07
AHES-17-II (delusions or thought insertion)		0.006	0.021	0.049	0.27	0.79
SPQ-B	Int	−0.016	0.029	−0.086	−0.55	0.59
	Cog	0.119	0.050	0.423	2.38	0.02^*^
	Dis	−0.107	0.038	−0.536	−2.78	0.01^*^
BDI-II		0.016	0.009	0.279	1.72	0.09

* < 0.05.

Table 2 also presents the correlations under each emotional condition or correlations between the false-alarm rates for non-critical lures and the scores on questionnaires. Regarding emotional valence, the correlation between LSHS and *d*′ was significant under the neutral (*r* = −0.29, *p* = 0.046) and positive (*r* = −0.29, *p* = 0.047) conditions, but not under the negative condition (*r* = −0.05, *p* = 0.74) (see the next section for the detailed analysis of emotional valence effect).

On the other hand, correlations between the total rate of false alarms for non-critical lures (regardless of emotional valence) and scores were not significant, which confirmed that even individuals in the current sample who were relatively high in terms of schizotypal traits had no semantic activation deficit. However, we found a significant correlation between LSHS (as well as AHES-17) scores and the rate of false alarms for non-critical lures under the negative condition (*r* = 0.31, *p* = 0.03). This result indicates that high-AHp subjects showed higher false-alarm rates for non-associated words under the negative condition (see the Discussion for a detailed interpretation). As the LSHS is used to measure auditory hallucination proneness in nonclinical samples (Larøi, 2012), and the LSHS was the strongest predictor of *d*′ in the current result, we focused on LSHS scores in the further detailed analyses.

### Proneness to auditory hallucinations and the effect of emotional valence

To directly investigate the relationship between the positive and negative emotional valence effect on the DRM task and AHp, we first divided participants into a low-AHp group and a high-AHp group based on LSHS scores (Table 5). Subjects who scored in the bottom 25^th^ percentile comprised the low-AHp group (*N* = 13), and those who scored in the upper 25^th^ percentile comprised the high-AHp group (*N* = 12) (see Table 5). Our cut-off point for bottom 25^th^ percentile was 18 (20 in Sugimori et al., 2011b) and the point of upper 25^th^ percentile was 29 (32 in Sugimori et al., 2011b). Our points for grouping are slightly lower than previous studies, but each group has nearly ten points gap between two groups. Furthermore, the following statistical results were identical when we divided participants using the criteria of +1 *SD* (*N* = 9) and −1 *SD* (*N* = 11).

**Table 5:** **Characteristics of low-and high-AHp groups**.

	**low-AHp**	**high-AHp**	**Significance**
Age	19.85 (1.95)	19.08 (0.67)	*p* = 0.21
Sex ratio (M/F)	12/1	8/4	
LSHS total score	15.15 (1.73)	32.75 (3.31)	*p* < 0.01^**^
AHES-17	37.54 (8.75)	57.42 (6.22)	*p* < 0.01^**^
SPQ-B	6.92 (4.91)	11.46 (3.05)	*p* = 0.01^*^
Cog	1.00 (1.08)	2.46 (1.57)	*p* = 0.01^*^
Int	3.77 (2.49)	5.27 (0.97)	*p* = 0.09
Dis	2.15 (2.15)	3.73 (1.74)	*p* = 0.07
BDI-II	9.31 (8.48)	11.58 (7.94)	*p* = 0.50

* < 0.05,

** < 0.01.

**Figure 4:**
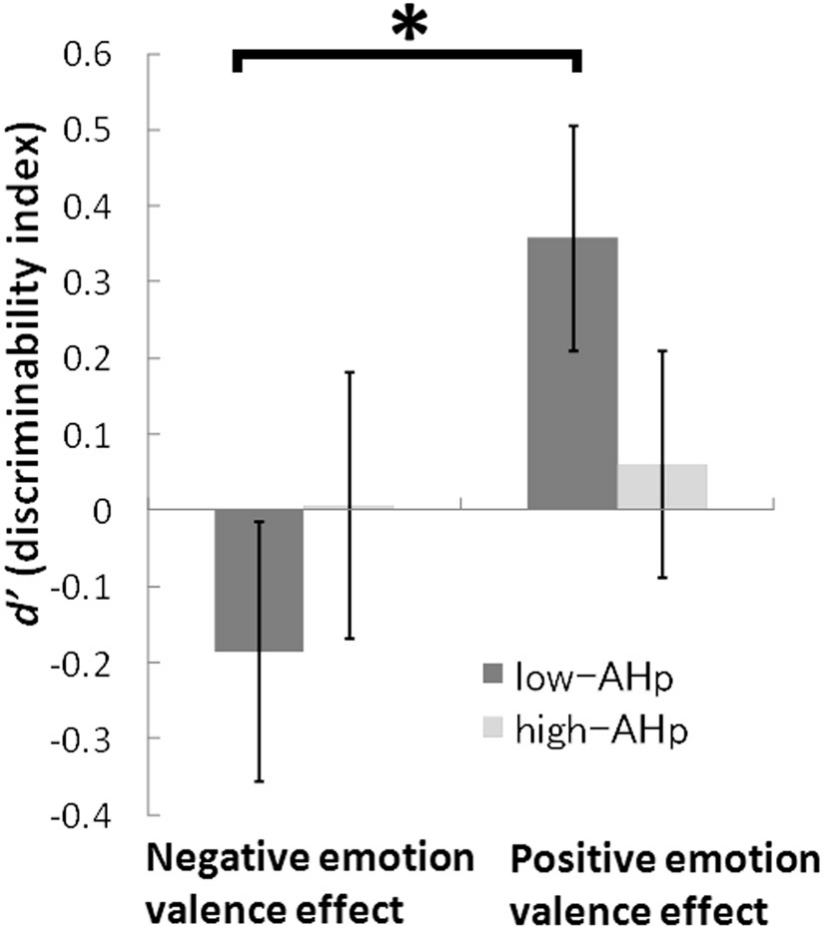
**Negative and positive emotional valence effect in AHp groups.** Note: negative emotion valence effect = negative − neutral condition. Positive emotion valence effect = positive − neutral condition. Error bars mean ± 1 S.E. ^*^ < 0.05.

Figure 4 shows the negative and positive emotional effects in the low-AHp and high-AHp groups. The negative effect was the difference between the negative and neutral conditions: negative-*d*′ - neutral-*d*′. The positive effect was the difference between the positive and neutral conditions: positive-*d*′ - neutral-*d*′. These calculations were necessary because the neutral-*d*′ was correlated with LSHS scores (Table 2), indicating that the neutral-*d*′ should be the baseline for the emotion effects. Whereas the low-AHp group showed similar emotion effects as found for the general population (Figure 1), the high-AHp group did not show these effects. A two-way ANOVA (two groups × two emotional valence effects) with repeated measures showed that the interaction between group and emotional valence effects [*F*_(1, 23)_ = 7.74, *p* = 0.01] and the main effect of emotional valence were significant [*F*_(1, 23)_ = 11.52, *p* = 0.002]. The simple main effect of emotional valence was also significant for the low-AHp group (*p* < 0.001), whereas the effect of emotional valence was not significant for the high-AHp group (Figure 4). These findings indicated that low-AHp participants showed positive and negative emotional valence effects in the DRM paradigm, whereas the high-AHp group did not show effects for either valence.

## Discussion

This study investigated the external misattribution of internally generated thoughts and emotional valence effects on it in high-AHp people. We used the LSHS and the AHES to measure auditory hallucination proneness, and a memory task, the DRM paradigm (Roediger and McDermott, 1995), to measure externalizing bias of internal thought. The DRM paradigm induces spontaneous thought of words semantically associated with presented words, and these thought of words are externalized with a high probability. If the self-monitoring hypothesis is true, then high-AHp people will make this error more often than low-AHp people. The present results showed that the LSHS score (measure of AHp in this study) was positively correlated with the overall *d*' results for critical lures (the total of negative, positive, and neutral lures). Additionally, we found no emotional valence effects among high-AHp subjects, even though these effects were observed in the low-AHp group. We discuss these results from the perspectives of both the self-monitoring theory for auditory hallucinations and the dual-process activation-monitoring framework in the DRM paradigm (McDermott and Watson, 2001).

### General performance of the emotional DRM paradigm

According to Figure 1, the emotional valence effect was reflected in data showing that the discriminability of negative words was lower than that for positive words. Previous studies have not observed the emotional valence effects in false memory in a consistent way (Dehon et al., 2010). However, recent studies have reported a higher frequency of false memories under the negative condition compared with the neutral condition when vigilance, word length, semantic similarities, and familiarity were controlled (Brainerd et al., 2008; Dehon et al., 2010). The results of the present study suggest that negative emotional valence increased false memories, which supports the results reported by Dehon et al. (2010) and Brainerd et al. (2008).

Brainerd et al. (2008) explained that the reason that the emotional valence effect increased false memories under the negative condition and decreased them under the positive condition was that similarities between false and true items increased under the negative condition whereas they decreased under the positive condition, thereby rendering confusion of true and false memories more likely under the negative than the positive condition. On the other hand, Dehon et al. (2010) suggested that the Paradoxical Negative Emotion (PNE) hypothesis was relevant to these results. The PNE hypothesis, recently put forward by Porter and colleagues (e.g., Porter et al., 2008, 2010) suggests that evolution has changed the brain such that negative information, compared with neutral or positive information, is more likely to be recalled over time and is also more vulnerable to memory distortions. According to the PNE hypothesis, there is adaptive value in having access to information from various sources regarding potential future dangers (Schacter and Addis, 2007); this increases the likelihood that people will misattribute internal negative content to actual external events. In summary, we not only increase semantic network activation by increasing semantic similarity, but also make more source-monitoring errors leading to experiencing more false memories in the DRM paradigm under the negative condition.

### Proneness to auditory hallucinations and external misattribution

As predicted, we replicated the positive correlation between AHp and the external misattribution of critical lures in the DRM task (Sugimori et al., 2011b). The same result was confirmed by multiple regression analysis. We conclude that this overall result (the total of the negative, positive, and neutral lures) indicates that high-AHp people tend to make more external misattributions of their internal thoughts given that no semantic activation deficits were found for participants in the present study. Moreover, this correlation persisted even when we controlled for the influence of negative mood (using the BDI-II). Apart from negative mood, which is commonly seen in people experiencing hallucinations, AHp relates to the external misattribution of internal thoughts. These results are important to future explorations of the mechanisms underlying auditory hallucinations, particularly in view of the fact that external misattribution of internal thoughts is said to be one form of thought insertion (Morrison et al., 1995). Many previous studies found a relationship between AHp and the external misattribution of actions and speech (e.g., Sugimori et al., 2011a; Asai and Tanno, 2012), but a relationship involving internally generated thoughts has not been reported until recently (Brébion et al., 2010; Sugimori et al., 2011b).

In this study, correlation and multiple regression analysis revealed that only the LSHS has a significant relationship with the performance of the DRM task. Interestingly, Sugimori et al. (2011b) reported a significant correlation between the performance of the DRM task and the AHES-17 and LSHS, and significant contribution of the AHES-17. To explore this issue further, we conducted a linear multiple regression analysis considering each of the two factors of the AHES-17 and the LSHS (Fonseca-Pedrero et al., 2010; Asai et al., 2011). We found that only the factor of vivid mental events in the LSHS made a significant contribution to experimental performance (Table 4). Many previous studies have reported that vividness of mental image and auditory hallucinations have a strong relationship (e.g., Van de Ven and Merckelbach, 2003). The present result suggests that vividness of mental image might have a stronger relationship with performance than auditory hallucination itself in the emotional DRM paradigm. It is still uncertain which components of psychotic phenomena are related to an externalizing bias. For example, Brookwell et al. (2013) reported that extant studies have found that an externalizing bias is related to hallucinatory predisposition only. However, delusional ideation has also been related to an externalizing bias (Dehon et al., 2008). Further research is needed to clarify this issue.

Regarding emotional valence, LSHS scores were significantly (*p* < 0.05) correlated with *d*′ under the neutral and positive conditions but not under the negative condition. Moreover, LSHS scores were significantly correlated with the false alarm-rates for non-critical lures under the negative condition (discuss in the next session). We have to note that these correlation results are exploratory and could be subject to Type I error. Because the lack of correlation under the negative condition cannot be understood within the context of source-monitoring theory, we examine this finding in the context of the dual-process considerations of the DRM paradigm in the section below.

### Proneness to auditory hallucinations and emotional deficits

We conducted a group comparison of high-AHp and low-AHp individuals to examine the potential effects of negative and positive emotional valence on the *d*′ for critical lures. We predicted two opposite results for the high-AHp group in terms of the effect of negative emotional valence on false memory in the DRM task. One prediction was that the external misattributions of critical lures would increase more in the high-AHp than in the low-AHp group under the negative emotional condition, possibly as a result of source-monitoring errors, which would be consistent with findings of earlier studies (Johns et al., 2001; Larøi et al., 2004; Costafreda et al., 2008). The second prediction was for minimal external misattributions of critical lures by the high-AHp group under the negative condition as participants in this group may not rely as heavily as those in the low-AHp group on emotion-driven associations for the activation of the semantic network in the DRM task. Our findings support the second prediction and, in terms of performance on the DRM task, the high-AHp group did not show negative or positive emotional valence effects. However, the low-AHp group revealed significant emotional valence effects such that the *d*′ under the negative condition was low and that under the positive condition was high under the neutral condition when compared with the comparable figures for the low-AHp group (Figure 4).

In healthy people, emotional valence strengthens associations between words because of emotional similarities (Kensinger, 2004). The high-AHp group, however, may not have made these emotion-driven semantic associations, perhaps because they might be not good at processing emotional components in a situation in which the auditory stimuli are presented very fast. Indeed, research has indicated that patients with schizophrenia experience delayed processing of emotional components compared with control subjects (Rockstroh et al., 2006; Seok et al., 2006). It has also been reported that schizotypal individuals are less affected by emotional priming, which implies they make fewer emotion-driven associations (Kerns, 2005). It is possible that the high-AHp group was unable to successfully make emotion-driven associations because of these deficits, given that the interval between words was very short in this study (about 300 ms). Moreover, studies have found that patients with schizophrenia experiencing positive symptoms tend to avoid directing attention at negative emotional stimuli because they are easily overwhelmed by the negative emotional components of these phenomena (Aleman and Kahn, 2005; Kerns, 2005; Seok et al., 2006). Thus, they may make fewer associations and retain verbatim memories by avoiding attending to negative images. This may also explain the correlation between LSHS scores and false-alarm rates for non-critical lures under the negative condition. That is, high-AHp subjects made more task-irrelevant mistakes as a result of being overwhelmed by negative words. Although the high-AHp group may have demonstrated more external misattribution of internal thoughts under the negative condition than did the low-AHp group, this effect may be offset by fewer emotion-driven associations.

The positive emotional valence effect in the low-AHp group (higher *d*′) may be attributable to decreased external misattribution and decreased semantic similarity. The PNE hypothesis holds that positive emotional valence decreases memory distortion (Porter et al., 2008). The high-AHp group may not have experienced this effect because of their tendency toward external misattribution and their deficits related to processing emotional components. However, further research into the positive emotional valence effect on external misattributions is needed.

### Limits of the present study and future issues

The present study has three limitations. First, it is still unclear which factor of the auditory hallucination experiences could be related to the externalizing bias. Despite the strong correlation between scores on the AHES-17 and those on the LSHS (*r* = 0.69) found in the present study, scores on the AHES-17 revealed no significant correlation with the performance of the DRM paradigm. Moreover, our analysis of each of the two factors of the LSHS and AHES-17 revealed that only the factor of vivid mental events made a significant contribution to the externalizing bias index. Future studies need to selectively use questions about auditory hallucination, delusional ideation, and vivid mental events. Second, the DRM lists used in the present study were not controlled in terms of vigilance or degree of semantic associations between emotional conditions. Thus, the results of this study related to emotional valence are related to the specific lists used. Previous studies that controlled for vigilance (e.g., Brainerd et al., 2008), degree of associations, familiarity, and word length (e.g., Dehon et al., 2010) also reported that false-alarm rates under the negative condition were higher than those under other conditions. In this context, the results of the present study may nonetheless suggest an emotional valence effect in the DRM paradigm and individual differences in AHp. Third, our sample size (i.e., 49) was smaller than that in other studies comparing high and low hallucination proneness (Badcock et al., 2008; Paulik et al., 2008). For example, Paulik et al. (2008) initially recruited over 500 participants to be assigned to either a high or a low LSHS group (final group size was 28 and 25 for each). Our sample data, however, has a distribution that is similar to that of the previous studies that utilized a large sample (172 in Sugimori et al., 2011b; 589 in Paulik et al., 2006). Further, our cut-off points for the high and low groups have a scoring gap between the two groups that is similar to the one found in Sugimori et al. (2011b). These data suggest that our high or low groups are directly comparable to those of previous studies.

This study found that high-AHp individuals showed a less pronounced emotional valence effect on false memories in the DRM paradigm than did low-AHp individuals. Moreover, when it comes to its responsible factor, the component of vividness of mental image in auditory hallucination proneness was most related to externalizing bias measured by the emotional DRM paradigm. In the future, we need only to develop a paradigm to enable separate examinations of the emotional valence effect on external misattributions and on semantic network activation in high-AHp individuals, and to further explore what component of psychotic symptoms is related to externalizing bias.

The English in this document has been checked by at least two professional editors, both native speakers of English. For a certificate, please see: http://www.textcheck.com/certificate/m0IdNq

### Conflict of interest statement

The authors declare that the research was conducted in the absence of any commercial or financial relationships that could be construed as a potential conflict of interest.
